# Supplementary material for: Prevalence and determinants of non-fistulous urinary incontinence among Ghanaian women seeking gynaecologic care at a teaching hospital
Source: PLoS One. 2020 Aug 18;15(8):e0237518. doi: 10.1371/journal.pone.0237518 (PMC7433879; doi:10.1371/journal.pone.0237518)
Supplement: S1 Table — (DOCX) [file pone.0237518.s001.docx]

## PREVALENCE OF URINARY INCONTINENCE QUESTIONNAIRE

**SOCIO-DERMOGRAPHIC CHARACTERISTICS**

| NO. | QUESTION AND FILTERS | RESPONSES | CODE | SKIP |
| --- | --- | --- | --- | --- |
| 1 | INITIALS/CODE |  |  |  |
| 2 | AGE(YEARS) |  |  |  |
| 3 | REASONS FOR REFERRAL |  |  |  |
| 4 | MARITAL STATUS | SINGLE  MARRIED  DIVORCED  CO-HABITING  WIDOW | 0  1  2  3  4 |  |
| 5 | EDUCATIONAL STATUS | NIL  PRIMARY/MSLC  JHS  SHS  TERTIARY  OTHER(SPECIFY) | 0  1  2  3  4  5 |  |
| 6 | OCCUPATION | Unemployed  Student  artisan(hairdresser,dressmaker etc)  trader/businessman  civil/public servant  teacher  farmer  health worker  other (specify) | 0  1  2  3  4  5  6  7  8 |  |
| 7 | HEIGHT |  |  |  |
| 8 | WEIGHT |  |  |  |
| 9 | BMI |  |  |  |

**OBSTETRIC AND GYNAECOLOGIC HISTORY**

| No | QUESTIONS AND FILTERS | RESPONSES | CODE | SKIPS |
| --- | --- | --- | --- | --- |
| 10. | GRAVIDITY |  |  |  |
| 11. | PARITY |  |  |  |
| 12. | MODE OF DELIVERY |  |  |  |
|  | 1^ST^ DELIVERY | SVD  VACUUM/FORCEPS  C/S | 0  1  2 |  |
|  | 2^ND^ DELIVERY | SVD  VACUUM/FORCEPS  C/S | 0  1  2 |  |
|  | 3^RD^ DELIVERY | SVD  VACUUM/FORCEPS  C/S | 0  1  2 |  |
|  | 4^TH^ DELIVERY | SVD  VACUUM/FORCEPS  C/S | 0  1  2 |  |
|  | 5^TH^ DELIVERY | SVD  VACUUM/FORCEPS  C/S | 0  1  2 |  |
|  | 6^TH^ DELIVERY | SVD  VACUUM/FORCEPS  C/S | 0  1  2 |  |
|  | 7^TH^ DELIVERY | SVD  VACUUM/FORCEPS  C/S | 0  1  2 |  |
|  | 8^TH^ DELIVERY | SVD  VACUUM/FORCEPS  C/S | 0  1  2 |  |
|  | 9^TH^ DELIVERY | SVD  VACUUM/FORCEPS  C/S | 0  1  2 |  |
|  | 10^TH^ DELIVERY | SVD  VACUUM/FORCEPS  C/S | 0  1  2 |  |
| 13 | EVER HAD A PERINEAL INJURY ? | NO  EPISIOTOMY  SPONTANEOS TEAR | 0  1  2 |  |
| 14. | ARE YOU MENOPAUSAL? | NO  YES | 0  1 |  |
| 15. | HAVE YOU HAD A SURGERY TO REMOVE YOUR WOMB? | NO  YES  NOT SURE | 0  1  2 |  |
| 16. | EVER HAD A VAGINAL SURGERY (FOR PROLAPSE OR FISTULA) | NO  YES  NOT SURE | 0  1  2 |  |

**PERSONAL HABITS**

| NO | QUESTIONS AND FILTERS | RESPONSES | CODE | SKIPS |
| --- | --- | --- | --- | --- |
| 17. | DO YOU SMOKE? | YES  NO | 0  1 |  |
| 18. | DO YOU DRINK ALCOHOL? | YES  NO | 0  1 |  |
| 19. | HOW OFTEN DO YOU DRINK TEA OR COFFEE? | NIL  <= ONE CUP/WEEK  ONE CUP/DAY  2-4 CUPS/DAY  5 OR MORE CUPS/DAY | 0  1  2  3  4 |  |
| 20. | HOW OFTEN DO YOU TAKE CARBONATED  DRINKS (e.g. COCA-COLA, SPIRITE ETC)? | NIL  <= ONE BOTTLE/WEEK  ONE BOTTLE/DAY  2-4 BOTTLES/DAY  5 OR MORE BOTTLES/DAY | 0  1  2  3  4 |  |

**MEDICAL HISTORY**

| NO | QUESTIONS AND FILTERS | RESPONSES | CODE | SKIPS |
| --- | --- | --- | --- | --- |
| 21. | DO YOU HAVE DIABETES MELLITUS? | YES  NO  NOT SURE | 0  1  2 |  |
| 22. | how often do you move your bowel in a week | once a week  Twice a week  Three times a week  More than 3 times a week | 0  1  2  3 |  |
| 23. | Have you experienced cough lasting more than 8 weeks in the last one year | YES  NO | 0  1 |  |

**SCREENING QUESTION FOR URINARY INCONTINENCE**

| NO | QUESTIONS AND FILTERS | RESPONSES | CODE | SKIPS |
| --- | --- | --- | --- | --- |
| 24. | HAVE YOU EXPERIENCED ANY INVOLUNTARY LOSS OF URINE IN THE PAST 4 WEEKS? | YES  NO | 1  2 | IF “YES” COMPLETE QUESTION 25 TO 28.  IF ”NO” END OF INTERVIEW |

**INTERNATIONAL CONSULTATION ON INCONTINENCE QUESTIONNAIRE-SHORT FORM**

| NO | QUESTIONS AND FILTERS | RESPONSES | CODE | SKIPS |
| --- | --- | --- | --- | --- |
| 25. | HOW OFTEN DO YOU LEAK URINE? | ABOUT ONCE A WEEK OR LESS  TWO TO THREE TIMES A WEEK  ABOUT ONCE A DAY  SEVERAL TIMES A DAY  ALL THE TIME | 0  1  2  3  4 |  |
| 26. | HOW MUCH URINE DO YOU USUALLY LEAK (WHETHER YOU WEAR PROTECTION OR NOT)? | A SMALL AMOUNT  MODERATE AMOUNT  LARGE AMOUNT | 0  1  2 |  |
| 27. | OVERALL, HOW MUCH DOES LEAKING URINE INTERFERE WITH YOUR EVERYDAY LIFE?  PLEASE CHOSE A NUMBER BETWEEN 0(NOT AT ALL) AND 10(A GREAT DEAL) | 0  1  2  3  4  5  6  7  8  9  10 | 0  1  2  3  4  5  6  7  8  9  10 |  |

**INTERNATIONAL CONSULTATION ON CONTINENCE-SHORT FORM (CONT’D)**

| 28. | WHEN DOES URINE LEAK? PLEASE TICK ALL THAT APPLY TO YOU | 1. LEAKS BEFORE YOU GET TO THE TOILET  2. LEAKS WHEN YOU COUGH OR SNEEZE  3. LEAKS WHEN YOU ARE ASLEEP  4. LEAKS WHEN YOU ARE PHYSICALLY ACTIVE/EXERCISING  5. LEAKS WHEN YOU HAVE FINISHED URINATING AND ARE DRESSED  6. LEAKS FOR NO OBVIOUS REASON  7. LEAKS ALL THE TIME | 0  1  2  3  4  5  6 |  |
| --- | --- | --- | --- | --- |

**THANK YOU VERY MUCH FOR ANSWERING THESE QUESTIONS**
