# Supplementary material for: Prevalence and determinants of non-fistulous urinary incontinence among Ghanaian women seeking gynaecologic care at a teaching hospital
Source: PLoS One. 2020 Aug 18;15(8):e0237518. doi: 10.1371/journal.pone.0237518 (PMC7433879; doi:10.1371/journal.pone.0237518)
Supplement: S1 File — (PDF) [file pone.0237518.s002.pdf]

Initial number

CONFIDENTIAL

DAY MONTH YEAR

Today's date

Many people leak urine some of the time. We are trying to find out how many people leak urine, and how much this bothers them. We would be grateful if you could answer the following questions, thinking about how you have been, on average, over the PAST FOUR WEEKS.

1 Please write in your date of birth:

DAY MONTH YEAR

2 Are you *(tick one)*:

Female ☐ Male ☐

3 How often do you leak urine? *(Tick one box)*

never	☐	0
about once a week or less often	☐	1
two or three times a week	☐	2
about once a day	☐	3
several times a day	☐	4
all the time	☐	5

4 We would like to know how much urine <u>you think</u> leaks.
How much urine do you <u>usually</u> leak (whether you wear protection or not)?
(Tick one box)

none	☐	0
a small amount	☐	2
a moderate amount	☐	4
a large amount	☐	6

5 Overall, how much does leaking urine interfere with your everyday life?
Please ring a number between 0 (not at all) and 10 (a great deal)

0 1 2 3 4 5 6 7 8 9 **10**

not at all — a great deal

ICIQ score: sum scores 3+4+5

6 When does urine leak? *(Please tick all that apply to you)*

never – urine does not leak	☐
leaks before you can get to the toilet	☐
leaks when you cough or sneeze	☐
leaks when you are asleep	☐
leaks when you are physically active/exercising	☐
leaks when you have finished urinating and are dressed	☐
leaks for no obvious reason	☐
leaks all the time	☐

Thank you very much for answering these questions.

Copyright © "ICIQ Group"

SAMPLE
