# Supplementary material for: Prevalence and determinants of non-fistulous urinary incontinence among Ghanaian women seeking gynaecologic care at a teaching hospital
Source: PLoS One. 2020 Aug 18;15(8):e0237518. doi: 10.1371/journal.pone.0237518 (PMC7433879; doi:10.1371/journal.pone.0237518)
Supplement: Attachment — Submitted filename: RESPONSE TO REVIEWERS.pdf [file pone.0237518.s004.pdf]

The Editor

PLOS | ONE

21/05/2020

Dear Sir,

**<u>REBUTTAL LETTER</u>**

***<u>MANUSCRIPT ID</u>***<u>:      PONE-D-20-00947</u>
***<u>MANUSCRIPT TITLE</u>***<u>: "PREVALENCE AND DETERMINANTS OF NON-FISTULOUS URINARY INCONTINENCE AMONG GHANAIAN WOMEN SEEKING GYNAECOLOGIC CARE AT A TEACHING HOSPITAL".</u>

We would like to express our sincere gratitude to the editors and the reviewers for their generous comments on our manuscript.

We have revised the manuscript to address all the concerns raised by the editors and the reviewers. Our responses are below and colour coded as follows;

1. Comments from editors/reviewers are shown as text in black
2. Our responses are shown as text in red.

We believe the manuscript is now suitable for publication in PLOS ONE.

Thank You

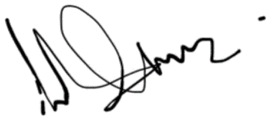

Dr Anthony Amanfo Ofori

Corresponding Author

**EDITORS/REVIEWERS COMMENTS AND RESPONSES**

***ACADEMIC EDITOR***

1, Referring to lines 71 to 73, can you rephrase? I think that I understand that you mean: (NF)UI is more studied in industrialized countries but I may be wrong.

Authors response: we have rephrased the statements to remove all ambiguities. The statement now reads "Non-fistulous urinary incontinence has been studied extensively in Europe, China and United States of America".

2, Please ensure that your manuscript meets PLOS ONE's style requirements, including those for file naming.

Authors response: All supporting files have been named according to the editorial requirements of PLOS ONE's journal as

"S1_Fig.tif

S2_Fig.tif

S3_Fig.tif

S1_Table.docx

S1_File.pdf

S2_File.dta"

3, In your Methods section, please provide additional information about the participant recruitment method and the demographic details of your participants.

Please ensure you have provided sufficient details to replicate the analyses such as:

a) the recruitment date range (month and year),

b) a description of any inclusion/exclusion criteria that were applied to participant recruitment and

c) a description of how participants were recruited.

Authors response: Additional details about recruitment period, inclusion and exclusion criteria and recruitment procedures have been provided in the methodology section of the manuscript. The following paragraph;

"All women attending the gynaecology clinic during the study period were assessed for eligibility by midwives who were research assistants and trained on questionnaire administration with its translation into the local dialect (Asante Twi). Study objectives were explained to participants and written informed consent obtained. Exclusion criteria comprised i) declining to give consent, ii) age below 18 years, iii) pregnancy at presentation, iv) women in the puerperium during the study period and v) women with a referral diagnosis of fistulous incontinence during the study period".

as written in the original manuscript has been rewritten in the revised manuscript as

"Between 1$^{st}$ January 2015 and 31$^{st}$ March 2015, all women accessing care at the gynaecology clinic irrespective of presenting complaints were assessed for eligibility by research assistants who were midwives working at the gynaecology clinic. Women below 18 years of age, women in their puerperium and those with a referral diagnosis of fistulous incontinence were excluded from the study at this point. The objectives of the study were explained to those eligible to partake in the study prior to consulting their physicians by the research assistants who have been trained to administer the questionnaires in English and the local (Asante Twi) language.

Written informed consent were obtained from those willing to participate in the study and the questionnaire administered to them by the research assistants

Inclusion criteria comprised All women who assessed care at the gynaecology clinic irrespective of presenting complaints during the study period and consented to participate in the study.

Exclusion criteria comprised i) women declining to give consent ii) women below 18 years of age iii) women with confirmed pregnancy during the study period iv) women in their puerperium during the study period v) women with a referral diagnosis of fistulous incontinence during the study period vi) women responding ‘NO’ to the screening question “have you experienced involuntary leakage of urine in the past one month” were excluded from completing the ICIQ-SF(the second part of the questionnaire).”

4. Thank you for including your ethics statement:

'Ethical clearance was obtained from the Committee for Human Research, Publication and Ethics (CHRPE) of the Kwame Nkrumah University of Science and Technology (CHRPE/AP/239/14).'

a. Please amend your current ethics statement to confirm that your named institutional review board or ethics committee specifically approved this study.

b. Once you have amended this/these statement(s) in the Methods section of the manuscript, please add the same text to the “Ethics Statement” field of the submission form (via “Edit Submission”).

Authors response: The statement has been amended to read “The study was approved by the committee for Human Research, Publication and Ethics (CHRPE) of the Kwame Nkrumah University of Science and Technology (CHRPE/AP/239/14)” and same has been added to the “Ethics Statement” field of the submission form.

5, Please include captions for your Supporting Information files at the end of your manuscript, and update any in-text citations to match accordingly.

Authors response: Caption for supporting information files has been added at the end of the manuscript and all supporting information files listed in accordance with the editorial requirements of PLOS ONE as shown below. In addition, all in-text citations have been updated to match accordingly.

## Supporting information

S1 Fig. **Profile of participant recruitment**

S2 Fig. **Referral diagnoses of study participants (%).**

S3 Fig. **Proportion of incontinent women by severity of incontinence (ICIQ score)**

S1 Table. **Prevalence of urinary incontinence questionnaire**

S1 File. **ICIQ-UI Short Form**

# S2 File. **prevalence of incontinence dataset**

REVIEWER #1

Thank you for submitting this manuscript

1. Please change urge incontinence to urgency incontinence

Authors response: Urge Incontinence has been changed to urgency incontinence in the manuscript.

2. No need to add a question to the validated questionnaire and you can simply put it in the exclusion criteria of patients.

Authors response: The point is noted. The manuscript has been revised with an addition to the exclusion criteria that states

“any participant that responded NO to the question {have you experienced any involuntary loss of urine in the past one month?} was excluded from completing the ICIQ-SF”

Subsequent to this modification, the phrase “modified version of ICIQ-SF”

has been revised to “ICIQ-SF”.

3. Why should you put the term non-fistulous in the title? You can actually mention the prevalence of urinary incontinence in the Ghana hospital.

Authors response: The term non-fistulous was added to highlight the point that the manuscript is not about fistulous incontinence which is more associated with obstructed labour and has been studied extensively in Ghana. We are of the opinion that using the term “non-fistulous” in the title will make the study easily identifiable if one is looking for such studies in Africa or Ghana.

REVIEWER #2

1. Methodology- highlight the inclusion criteria

Authors response: As suggested the manuscript has been revised to highlight the inclusion criteria with the inclusion of the statement

**“**Inclusion criteria comprised; All women who assessed care at the gynaecology clinic irrespective of presenting complaints during the study period and consented to participate in the study”.

2. Results- elaborate on the impact of urinary incontinence on the lifestyle, there should be a table for it

Authors response: The statement “On the overall interference with everyday life scale which ranged from 0 to 10 (with 0 representing no inconvenience and 10 representing a great deal of inconvenience), more than a half of the incontinent women (28/48, 58.3%) gave a score of 5 or more with 2% (1/48) giving a score of 0” has been reworded to read

“On the overall interference with everyday life scale which ranged from 0 to 10 (with 0 representing no inconvenience and 10 representing a great deal of inconvenience) only 2% (1/48) of incontinent women reported no interference with daily life. Approximately 31% (15/48) reported mild interference with daily life whiles about 42% of incontinent women said urinary incontinence moderately interfered with their daily life”. In addition, Table 2 with the caption “interference of urinary incontinence with everyday life of affected women.” has been inserted.

3. Reference- Reference 18 should be written properly

Authors response: Reference 18 has been revised accordingly. It now reads

“Bello OO (2018) Prevalence of Non-fistulous Urinary Incontinence Among Nonparturient Women in A Tertiary Hospital. Journal of Woman's Reproductive Health 2: 35”

instead of

“Bello OO. JOURNAL OF WOMAN’S REPRODUCTIVE HEALTH”. as was contained in the original manuscript.
