# Supplementary material for: Prevalence and determinants of non-fistulous urinary incontinence among Ghanaian women seeking gynaecologic care at a teaching hospital
Source: PLoS One. 2020 Aug 18;15(8):e0237518. doi: 10.1371/journal.pone.0237518 (PMC7433879; doi:10.1371/journal.pone.0237518)
Supplement: Attachment — Submitted filename: RESPOSNSE TO REVIEWERS.docx.pdf [file pone.0237518.s005.pdf]

The Editor

PLOS | ONE

17/07/2020

Dear Sir,

REBUTTAL LETTER

***MANUSCRIPT ID*: PONE-D-20-00947R1**
***MANUSCRIPT TITLE*: "PREVALENCE AND DETERMINANTS OF NON-FISTULOUS URINARY INCONTINENCE AMONG GHANAIAN WOMEN SEEKING GYNAECOLOGIC CARE AT A TEACHING HOSPITAL."**

We would like to express our sincere gratitude to the editors and the reviewers for their generous comments on our manuscript titled "PREVALENCE AND DETERMINANTS OF NON-FISTULOUS URINARY INCONTINENCE AMONG GHANAIAN WOMEN SEEKING GYNAECOLOGIC CARE AT A TEACHING HOSPITAL."

We have revised the manuscript to address all the concerns raised by the editors and the reviewers. We believe the manuscript is now suitable for publication in PLOS ONE journal. Please find below our responses to the issues raised.

Thank You

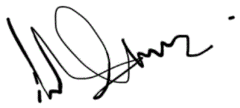

Dr Anthony Amanfo Ofori

Corresponding Author

RESPONSES TO REVIEWERS

- **ACADEMIC EDITOR**

1. Academic made the following comment;

"I would add that it is good to make clear in the discussion paragraphs that it is about women who visited the gynaecological clinic. Sometimes the conclusions seem to generalize too much, as if it is a population screening."

Response: The statement in the first paragraph of the discussion segment has been reworded to make it clear. The original statement read;

"This cross-sectional study was conducted to assess the prevalence of urinary incontinence among women, factors associated with its occurrence as well as its self-reported interference with everyday life activities of those affected" (line 289-291).

It now reads;

"This cross-sectional study was conducted to assess the prevalence of urinary incontinence among women visiting an out-patient gynaecologic clinic, determine factors associated with its occurrence as well as its self-reported interference with everyday life activities of those affected"

(line 288-291)

- **REVIEWER 3**

MAJOR

1. Reviewer 3 made the following extracted comment on line 137

"………and if the questionnaires were translated into Asanti Twe language. Also to inform the reader the reason why orally administered questionnaire was used versus written answered questionnaires."

Response : it has been clarified that the bulk of the questionnaires had to be orally administered by verbal translation from English into the local language because most respondents had little or no English reading and comprehension abilities. Only a handful were able to self-administer the English version but we cannot provide data on how many could self-administer since we did not count. It was not considered relevant to the work.

The portions of the manuscript reflecting the changes are indicated below……………..

Lines 136-139 originally read as;

“physicians by the research assistants who have been trained to administer the questionnaires in English and the local (Asante Twi) language. Written informed consent were obtained from those willing to participate in the study and the questionnaire administered by the research assistants”

It now reads as (136-141);

“physicians by research assistants who had been trained to administer the questionnaires. The vast majority of questionnaires were administered through verbal translation into the local Asante Twi language as most participants had little or no English reading and comprehension skills while only a handful were self-administered in English. Written informed consent were obtained from those willing to participate in the study.”

2. Reviewer 3 made the following comment on line 159

Author should consider to comment on the validity of the questionnaires used as a measuring instrument

Response: The questionnaire used (ICIQ-SF) has not been validated in the Ghanaian population. This would require a comparison of its output with urodynamic studies as a gold standard test with measurement of certain metrics. As earlier stated, this has not been done in the Ghanaian population and our study did not include it.
This has been clarified in the methodology and in the discussion

The portions of the manuscript reflecting the changes are indicated below……………..

Line 159-160 originally read as;

“This validated symptom questionnaire is made up of three scored questions…………………”

It now reads as (lines 161-162);

“This symptom questionnaire is made up of three scored questions………………..”

In addition, a new sentence has been introduced (lines 173-174) as;
“The ICIQ-SF, as used, has not been validated in the Ghanaian population.”

Furthermore, under the Discussion, the use of a measuring tool that has not been validated in the population has been acknowledged as a limitation and also put up for further research.

A new sentence has been introduced in the last paragraph of the DISCUSSION (line 362-364): "It is also possible the questionnaire underestimated the burden of urinary incontinence as it has not been validated previously in the Ghanaian population as done elsewhere"

ALSO, lines 366-367 originally reading as: "Further studies are needed to assess the health seeking behaviour of women with urinary incontinence" has now been modified to read as;

(Lines 370-372) "Further studies are needed to validate the ICIQ-SF tool against urodynamic studies in Ghana and to assess the health seeking behaviour of women with urinary incontinence"

3. Reviewer 3 made the following comment on line 357

Author claims parity was not associated with urinary incontinence but Table 4 indicates all of the population is parous. This will need explanation

Response: All the population was NOT parous as Table 1 shows 115 respondents with zero parity. Table 4 only shows Odds ratios and p-values for association (parity versus incontinence) for the parity groups 1-4 and ≥5 compared to the baseline of zero parity. We think therefore that line 357 should stand.

4. Reviewer 3 made the following comment on line 359

The author may consider to comment on limitations or strengths in the study:
i) if local validated questionnaire was used and
ii) written and self completed questionnaire versus orally administered questionnaire

Response:

i) Issues on the validity of the questionnaire and its implications have been addressed under No. 2

ii) The question of self-administered questionnaire versus orally administered questionnaire has been addressed under No. 1. Since the vast majority of

questionnaires were orally administered, limitations of selection bias arising from any disparity between the two routes is considered insignificant and will not impact negatively on the findings

A new sentence is introduced (lines 364-367) to reflect this stance as below;

“Any bias emanating from the different routes of questionnaire administration is considered negligible as the vast majority were orally administered and is not expected to adversely affect the study findings”

5. Reviewer 3 made the following comment on line 365

The author should indicate if the assumption to educate clinicians re screening for urinary incontinence is valid from this study or if it still requires further research

Response: We have elected to do away with the recommendation in question.

Hence, lines 365-366 which originally read as;

“Policy guidelines should aim at educating clinicians to screen for the condition in women attending gynaecology clinics” has been eliminated.

MINOR

1. The reviewer draws attention to a spelling error on line 170

Response:

The misspelt word “scrennning” has been changed to “screening” and is now on line 172

2. Reviewer 3 made the following comments on Reference 1.

   “To consider to use the most lately published article on Standardization of terminology”

   Response:

We have replaced the old reference

“Abrams P, Cardozo L, Fall M, Griffiths D, Rosier P, et al. (2003) The standardisation of terminology in lower urinary tract function: report from the standardisation sub-committee of the International Continence Society. Urology 61: 37-49.”

with the most lately published article on standardization of terminology.

“Haylen BT, De Ridder D, Freeman RM, Swift SE, Berghmans B, et al. (2010) An International Urogynecological Association (IUGA)/International Continence Society (ICS) joint report on the terminology for female pelvic floor dysfunction. Neurourology and Urodynamics: Official Journal of the International Continence Society 29: 4-20.”

The reference list has been updated appropriately.

3. Reviewer 3 made the following comments on reference 31.

   “This reference appears to be incomplete ... Could not access properly via Google scholar”.

   Response:

   We concede the point that the reference as appears in Google Scholar is incomplete. Unfortunately, we are not in a position to provide the additional information to make the reference complete at the moment. As a result, the old reference

   “ **Cardozo L (1997) Urogynecology: the King's approach: WB Saunders Company**.” has been replaced with a more recent referrence;

   “**Gomelsky A, Dmochowski RR (2011) Urinary incontinence in the aging female: etiology, pathophysiology and treatment options. Aging Health 7: 79-88**”.

   Subsequently the wording of that part of the manuscript has been amended as follows;

   Line 331-334 originally read as;

“It has been reported that aging predisposes individuals to urinary incontinence due to reduction in bladder capacity, decreased urethral closure pressure and decreased ability to delay voiding”…

It now reads (line 331-335)

“The aged may be prone to the development of urinary incontinence due to hypoestrogenism, decreased urethral closure pressure and the development of urodynamic detrusor overactivity... In addition impaired mobility and increased nocturnal urine production may contribute to the development of urinary incontinence”…

Thnak you
