# Supplementary material for: Prevalence and determinants of non-fistulous urinary incontinence among Ghanaian women seeking gynaecologic care at a teaching hospital
Source: PLoS One. 2020 Aug 18;15(8):e0237518. doi: 10.1371/journal.pone.0237518 (PMC7433879; doi:10.1371/journal.pone.0237518)
Supplement: Attachment — Submitted filename: RESPONSE TO REVIEWERS.docx [file pone.0237518.s006.docx]

**RESPONSE TO REVIEWER No. 3**

Reviewer #3: Thank you for your response.
You have answered the questions and made some changes to the text, but some of the changes does not reflect in your abstract. This needs to be corrected
I feel the reader needs to be clearly informed in the methodology section if the ICIQ-SF was translated prior to the interview to Ghanaian language but not validated, or if the person who introduced the questionnaire translated the questionnaire into Ghanaian language at the same time of the interview.

**Response:**

The authors appreciate the reviewer’s opinion and keen sense of detail in reviewing our work. Under Methods, It has been clarified that translation into the local language was done at the same time as the interview was being conducted.

The relevant section of the Methods section originally read as (lines 137-141);

“……..The vast majority of questionnaires were administered through verbal translation into the local Asante Twi language as most participants had little or no English reading and comprehension skills while only a handful were self-administered in English. Written informed consent were obtained from those willing to participate in the study. “

It has now been revised to read as (139-144);

“…….The vast majority of questionnaires were administered through verbal translation into the local Asante Twi language as most participants had little or no English reading and comprehension skills while only a handful were self-administered in English. Translation of the questionnaire into the local language was done at the same time of the interview. Written informed consent were obtained from those willing to participate in the study.”

**Additionally**, relevant changes have also been effected in the abstract to reflect reviewer comments.

**The abstract originally read as shown below;**

The study assessed the prevalence and determinants of non-fistulous urinary incontinence among gynaecologic care seekers as well as its interference with everyday life activities of affected women.

A cross-sectional study involving 400 women was conducted in a tertiary facility. Urinary incontinence was assessed using the International Consultation on Incontinence Questionnaire-short form (ICIQ-SF). The data was analysed for proportions and associations between selected variables.

The prevalence of urinary incontinence was 12%, the common types being urgency (33.3%), stress (22.9%), and mixed (20.8%). Age ≥60 years compared to 18-39 years (OR 3.66 95%CI 1.48-9.00 P=0.005), and a history of chronic cough (OR 3.80 95% CI 1.36-10.58 P=0.01) were associated with urinary incontinence. Women with education beyond the basic level were 72% less likely to experience urinary incontinence (OR 0.28 95%CI 0.08-0.96 P=0.04). Urinary incontinence interferes with everyday life activities of most affected women.

Non-fistulous urinary incontinence is relatively common among gynaecologic care seekers yet very few women were referred with such a diagnosis. Physicians should be educated to screen all women seeking gynaecologic care for incontinence with the aim of identifying women with such conditions for appropriate treatment.

**It has now been revised to read as;**

The study assessed the prevalence and determinants of non-fistulous urinary incontinence among gynaecologic care seekers as well as its interference with everyday life activities of affected women.

A cross-sectional study involving 400 women was conducted in a tertiary facility in Ghana. Urinary incontinence was assessed using the International Consultation on Incontinence Questionnaire-short form (ICIQ-SF) which has not been validated locally. The questionnaire was administered mostly in the Asante Twi language with translation done at the time of the interview. The data was analysed for proportions and associations between selected variables.

The prevalence of urinary incontinence was 12%, the common types being urgency (33.3%), stress (22.9%), and mixed (20.8%). Age ≥60 years compared to 18-39 years (OR 3.66 95%CI 1.48-9.00 P=0.005), and a history of chronic cough (OR 3.80 95% CI 1.36-10.58 P=0.01) were associated with urinary incontinence. Women with education beyond the basic level were 72% less likely to experience urinary incontinence (OR 0.28 95%CI 0.08-0.96 P=0.04). Urinary incontinence interferes with everyday life activities of most affected women.

Non-fistulous urinary incontinence is relatively common among gynaecologic care seekers yet very few women were referred with such a diagnosis. Advocacy measures aimed at urging affected women to report the condition and educating the general population on potential causes, prevention and treatment are needed.

**RESPONSE TO ACADEMIC EDITOR**

In agreement with the reviewer’s comments: Can you be slightly more precise in your methods, and adapt the abstract?

**Response:** The detail suggested by the reviewer has been included and the abstract revised to reflect all changes made to the body of the manuscript.

The relevant section of the Methods section originally read as (lines 137-141);

“……..The vast majority of questionnaires were administered through verbal translation into the local Asante Twi language as most participants had little or no English reading and comprehension skills while only a handful were self-administered in English. Written informed consent were obtained from those willing to participate in the study. “

It has now been revised to read as (139-144);

“…….The vast majority of questionnaires were administered through verbal translation into the local Asante Twi language as most participants had little or no English reading and comprehension skills while only a handful were self-administered in English. Translation of the questionnaire into the local language was done at the same time of the interview. Written informed consent were obtained from those willing to participate in the study.”

**Additionally**, relevant changes have also been effected in the abstract to reflect reviewer comments.

**The abstract originally read as shown below;**

The study assessed the prevalence and determinants of non-fistulous urinary incontinence among gynaecologic care seekers as well as its interference with everyday life activities of affected women.

A cross-sectional study involving 400 women was conducted in a tertiary facility. Urinary incontinence was assessed using the International Consultation on Incontinence Questionnaire-short form (ICIQ-SF). The data was analysed for proportions and associations between selected variables.

The prevalence of urinary incontinence was 12%, the common types being urgency (33.3%), stress (22.9%), and mixed (20.8%). Age ≥60 years compared to 18-39 years (OR 3.66 95%CI 1.48-9.00 P=0.005), and a history of chronic cough (OR 3.80 95% CI 1.36-10.58 P=0.01) were associated with urinary incontinence. Women with education beyond the basic level were 72% less likely to experience urinary incontinence (OR 0.28 95%CI 0.08-0.96 P=0.04). Urinary incontinence interferes with everyday life activities of most affected women.

Non-fistulous urinary incontinence is relatively common among gynaecologic care seekers yet very few women were referred with such a diagnosis. Physicians should be educated to screen all women seeking gynaecologic care for incontinence with the aim of identifying women with such conditions for appropriate treatment.

**It has now been revised to read as;**

The study assessed the prevalence and determinants of non-fistulous urinary incontinence among gynaecologic care seekers as well as its interference with everyday life activities of affected women.

A cross-sectional study involving 400 women was conducted in a tertiary facility in Ghana. Urinary incontinence was assessed using the International Consultation on Incontinence Questionnaire-short form (ICIQ-SF) which has not been validated locally. The questionnaire was administered mostly in the Asante Twi language with translation done at the time of the interview. The data was analysed for proportions and associations between selected variables.

The prevalence of urinary incontinence was 12%, the common types being urgency (33.3%), stress (22.9%), and mixed (20.8%). Age ≥60 years compared to 18-39 years (OR 3.66 95%CI 1.48-9.00 P=0.005), and a history of chronic cough (OR 3.80 95% CI 1.36-10.58 P=0.01) were associated with urinary incontinence. Women with education beyond the basic level were 72% less likely to experience urinary incontinence (OR 0.28 95%CI 0.08-0.96 P=0.04). Urinary incontinence interferes with everyday life activities of most affected women.

Non-fistulous urinary incontinence is relatively common among gynaecologic care seekers yet very few women were referred with such a diagnosis. Advocacy measures aimed at urging affected women to report the condition and educating the general population on potential causes, prevention and treatment are needed.
